# Computational medication regimen for Parkinson’s disease using reinforcement learning

**DOI:** 10.1038/s41598-021-88619-4

**Published:** 2021-04-29

**Authors:** Yejin Kim, Jessika Suescun, Mya C. Schiess, Xiaoqian Jiang

**Affiliations:** 1 School of Biomedical Informatics, University of Texas Health Science Center at Houston, 7000 Fannin St., Houston, TX USA; 2 Department of Neurology, McGovern School of Medicine, University of Texas Health Science Center At Houston, Houston, TX USA

**Keywords:** Parkinson's disease, Computational science, Computer science

## Abstract

Our objective is to derive a sequential decision-making rule on the combination of medications to minimize motor symptoms using reinforcement learning (RL). Using an observational longitudinal cohort of Parkinson’s disease patients, the Parkinson’s Progression Markers Initiative database, we derived clinically relevant disease states and an optimal combination of medications for each of them by using policy iteration of the Markov decision process (MDP). We focused on 8 combinations of medications, i.e., Levodopa, a dopamine agonist, and other PD medications, as possible actions and motor symptom severity, based on the Unified Parkinson Disease Rating Scale (UPDRS) section III, as reward/penalty of decision. We analyzed a total of 5077 visits from 431 PD patients with 55.5 months follow-up. We excluded patients without UPDRS III scores or medication records. We derived a medication regimen that is comparable to a clinician’s decision. The RL model achieved a lower level of motor symptom severity scores than what clinicians did, whereas the clinicians’ medication rules were more consistent than the RL model. The RL model followed the clinician’s medication rules in most cases but also suggested some changes, which leads to the difference in lowering symptoms severity. This is the first study to investigate RL to improve the pharmacological approach of PD patients. Our results contribute to the development of an interactive machine-physician ecosystem that relies on evidence-based medicine and can potentially enhance PD management.

## Introduction

**Figure 1:**
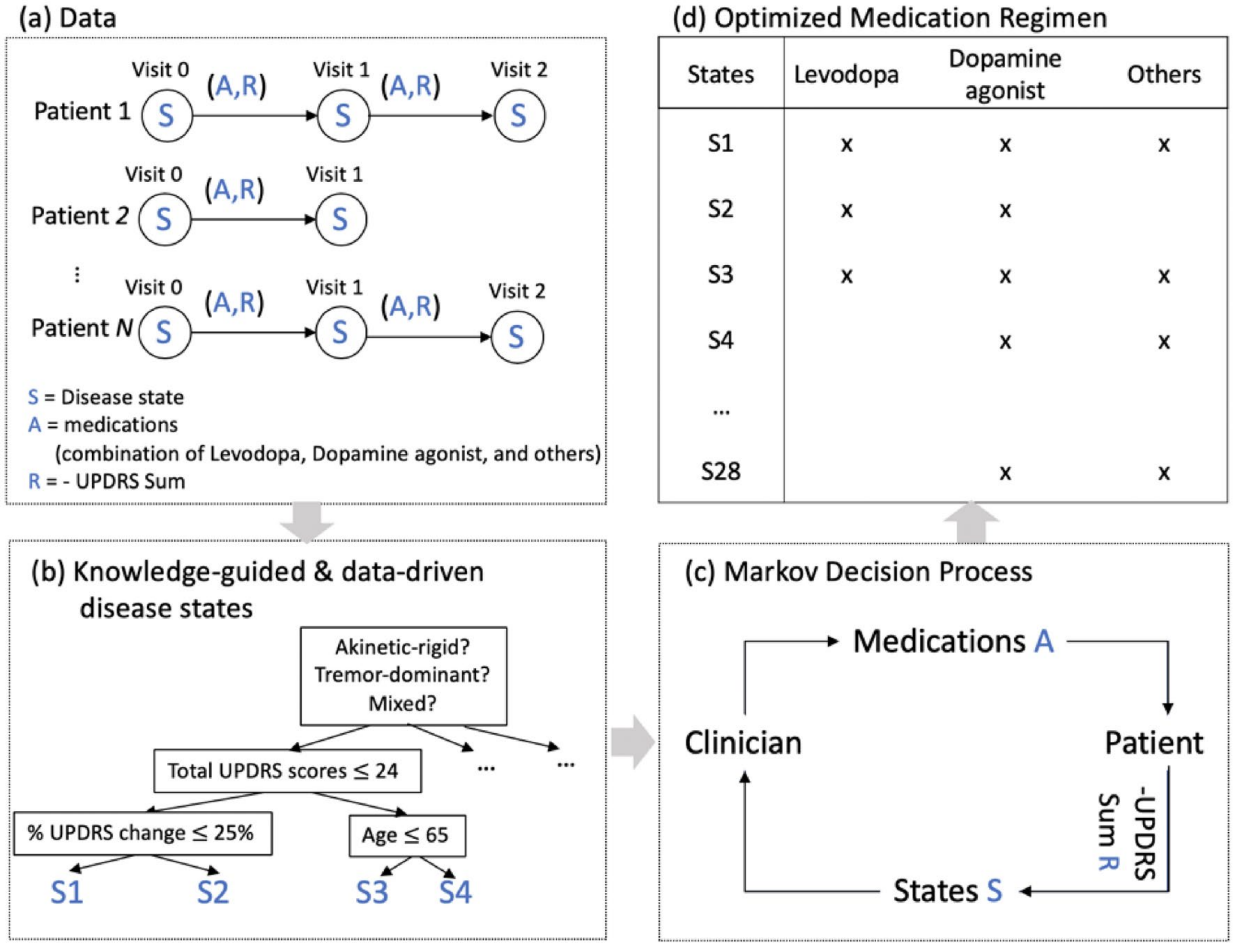
Workflow data from an optimal medication regimen. We aimed to derive an optimal medication regimen that suggests the best combination of drugs given the current disease state to minimize motor symptoms of PD. (**a**) PPMI database consists of trajectories of visits of PD patients. Each visit has clinical assessments to measure the current disease status and the record of the medications used to address the motor symptoms. UPDRS III score measures the motor response to the medication. (**b**) Each visit was characterized as one of the discrete disease states that are defined using a decision tree regressor. (**c**) We formulated this medication therapy as an MDP. Based on the current disease state, a clinician makes a decision on a combination of medications, which in turn change motor symptoms and alters the disease state iteratively. (**d**) We computed the optional medication option on each state using policy iteration (in reinforcement learning).

Parkinson’s disease (PD) is the fastest-growing neurodegenerative disorder and by 2040 it will become affecting 17.5 million people worldwide. Since 1990, the crude prevalence rate of this disease has increased by 74.3% and the US annual economic burden of PD is estimated at $52 billion^1^. PD treatment is primarily focused on symptomatic relief using dopamine replacement^2–4^. Pharmacological management represents a decision-making challenge that requires careful consideration of a number of factors that change as the disease progresses (Fig. 1a). PD is a complex and heterogeneous disorder^4^, and despite having treatment guidelines, there are multiple elements that need to be considered when deciding which intervention to choose (Fig. 1b). Clinicians usually start with a low dose monotherapy and later on gradually adjust the dosage and/or add an adjunct therapy depending on the patient's response and the disease state (Fig. 1c)^2,3^. This decision-making process has multiple steps that lead to the appropriate combination of medications (Fig. 1d) that address motor symptoms and improve quality of life. Large observational databases provide a unique opportunity to learn regimen optimization through past medication and the patient’s response, however, extracting knowledge from these prospective databases require more than human intuition alone^5^.

Reinforcement learning (RL) is an area of artificial intelligence (AI) and the third branch of machine learning (besides supervised and unsupervised learning) for optimizing sequential decision-making problems based on observed outcomes. It has shown great potential and outperformed humans in playing games like *Atari*^6^ and *Go*^7^. RL naturally models a sequential decision-making problem as a Markov decision process (MDP). An MDP consists of a set of *states*, *actions*, and *rewards* (Fig. 1c). A virtual agent in a certain *state* selects an *action* to maximize cumulative future *reward*. The agent explores a vast amount of actions (and their combinations) as a trial-and-error process to discover the next best action that yields the maximum instant reward and all the subsequent rewards after all. While they are still relatively new to medicine, RL technologies have been recently introduced into the clinical field, including the treatment of sepsis^8^, epilepsy^9^, lung cancer^10^, and propofol infusion administration^11^. RL can be used to model the real-world treatment process (instead of supporting the clinical decision from a single snapshot as typical machine learning models do) and potentially improve clinical practice by maximizing treatment outcomes, which aligns with the goals of evidence-based medicine^12^.

Most recently, Komorowski et al*.* has successfully shown the potential of RL models in medicine by using RL to find the best combination of medication dosage to reduce mortality rates in patients with sepsis^8^. However, two main concerns arise when considering utilizing the model in clinical practice: *interpretability* and *robustness*. Komorowski et al*.* define several hundreds of disease states using a purely data-driven approach (without incorporating prior knowledge on the disease) and although it incorporates a vast amount of available data, it loses interpretability and accessibility. This might be acceptable in an inpatient setting as there is an enormous quantity of information flowing into the system (still hard to interpret in real-time), but is not ideal for an outpatient setting like long-term management of PD. In addition, the sepsis RL study chose one of the best policies from several trials, which does not evaluate the robustness and generalizability of the model, making the model less likely to be executed in practice. Therefore, we aim to highlight the clinical usefulness by addressing the interpretability (using knowledge-embedded decision tree regressor) and robustness (using an ensemble technique). Our objective is to show the effectiveness of RL in deriving a robust and optimal combination of medication for each PD disease state. This is the first study to investigate RL to improve the management of a chronic condition like PD.

## Materials and methods

### Database

We built the sequential decision-making model based on the Parkinson’s Progression Markers Initiative (PPMI) database as a retrospective cohort study. PPMI is a large-scale international prospective observational study that started in 2010. Clinical assessments such as Hoehn and Yahr stage, Montreal Cognitive Assessment (MoCA), and the Unified Parkinson Disease Rating Scale (UPDRS) section III and medication (Levodopa, a dopamine agonist, other PD medications) are collected at every visit.

### Patient cohort

We selected 431 early-stage PD patients who have initial Hoehn and Yahr stage ≤ 2, UPDRS III motor scores, and complete medication history (Table 1). Patients usually visit every 2–3 months for follow up. We excluded patients who had only one visit. A total of 431 patients have 11.8 visits on average and a maximum of 16 visits. The follow-up period was 55.5 months on average and a maximum of 96 months. The total number of visits was 5077.

**Table 1:** Patient demographics.

Patients (n = 431)	
Age at onset, years (mean, s.d.)	61.7 (9.8)
Num. of visits (mean, s.d.)	11.8 (3.5)
Months of follow-up (mean, s.d.)	55.5 (26.6)
**Gender**	
Num. of female	149
Num. of male	282
Visit (n = 5077)	
Total UPDRS scores (mean, s.d.)	24.4 (11.6)
Total MoCA scores (mean, s.d.)	26.6 (3.0)
**Subtype**	
Akinetic-rigid type	4568
Tremor-dominant type	647
Mixed type	335
**Hoehn and Yahr stage**	
1	1476
2	3800
3	213
4	28
5	6
**Medication**	
No drugs	1839
Levodopa	1157
Dopamine agonist	447
Other PD medication	442
Levodopa + others	356
Levodopa + Dopamine agonist	333
Dopamine agonist + others	260
Levodopa + Dopamine agonist + others	243

*PD* Parkinson’s disease, *UPDRS* Unified Parkinson’s Disease Rating Scales Part III motor score.

### Medications and clinical outcomes

Most of the patients were taking a combination of levodopa, a dopamine agonist, and other PD medications (MAO-B inhibitors, COMT inhibitors, Amantadine, and Anticholinergics). There were ${2}^{3}$ combinations of medication with levodopa, a dopamine agonist, and other PD medications. Note that as an observational study, PPMI is not testing any medications or devices, and the study subjects are expected to receive the standard of care for PD. The medication effect on motor symptoms was measured as a function of the UPDRS section III in the “ON state”^13^.

### Clinical assessments

Clinical assessments at each visit included the 18 subitems of the UPDRS III motor examination, 30 subitems of MoCA, and the Hoehn and Yahr stage. We computed the % change of total UPDRS III scores to measure the rate of progression. That is, % change of total UPDRS III scores at time ${t}_{1}$ is defined as 100%* (Total UPDRS III scores at ${t}_{1}$-Total UPDRS III scores at ${t}_{0}$)/Total UPDRS III scores at ${t}_{0}$. A subtype was given to each visit based on the main cause of disability in motor function. The subtype was defined as either akinetic-rigid, tremor-dominant, or mixed type based on our previous study^14^.

### Medication therapy as a Markov decision process

Symptomatic medication management consists of three components: current disease state, medication options, and total UPDRS III scores. Based on the current disease state, a clinician makes a decision on medication combination. The medication combination affects the patient's motor symptoms and changes the current disease state iteratively. The clinician’s goal is to minimize current and future total UPDRS III scores by selecting the best medication combination.

We modeled this iterative medication therapy as an MDP, which is a framework to represent a sequential decision-making problem. In MDP, a virtual agent in a certain state selects actions to maximize future rewards. The agent explores a vast amount of actions as a trial-and-error to discover the best next action that yields the maximum instant reward and all the subsequent rewards. We formally rephrase medication therapy as:

- *State* $s$: A finite set of disease states at the current visit.
- *Action* $a$: The eight combination of drugs of Levodopa, a dopamine agonist, and other PD medications given at each visit (from no drugs given to all drugs given).
- *Reward/Penalty* $r(s,a,s{^{\prime}})$: Patient’s response to the medication. Clinician’s goal is to minimize symptom level (i.e., penalty), which was defined as the cumulative sum of total UPDRS III scores.

### Representing a patient’s current disease state

In order to formulate the medication therapy as MDP, the patient’s current disease state at every visit should be quantified as one of the discrete states. We used various features including PD subtypes, Hoehn and Yahr stage, age at the visit, MoCA score, total UPDRS III scores, % change of total UPDRS III scores, and previous medication (Table 2). We first validated that potential variables used in defining disease states and actions have statistical significance in predicting the next total UPDRS scores (penalty). Using the validated variables, we derived decision tree regressors to define the disease states. The decision tree regressor has advantages in deriving mutually exclusive and discrete states that have discriminability and ready-to-use interpretability in actual clinical practice. We enforced the first splitting rules as the main cause of disability (i.e., subtypes that are either akinetic-rigid, tremor-dominant or mixed type) according to clinical practice guidelines^2,3^. Then we found the best variables and their threshold to predict the next total UPDRS scores. We ensured the minimum number of visits that lying in the disease state to be no smaller than 100 to avoid too many fine-grained disease states without enough visits.

**Table 2:** Statistical significance of variables used in disease state definition (i.e., clinical assessments) and action (medications).

Time	Variables	Coef	*p*-value
${t}_{1}$	(Variables used in disease state definition)		
	Hoehn and Yahr	0.8967	0
	Age	0.0864	0
	Total MoCA	− 0.0534	0.01
	Total UPDRS III scores	0.7816	0
	% change of total UPDRS scores	− 0.0246	0
	Subtype AR (vs. mixed type)	1.5576	0
	Subtype TD (vs. mixed type)	0.6056	0.213
	Levodopa	5.5486	0
	Dopamine agonist	4.5439	0
	Other medicine	2.2017	0
	Levodopa + Other medicine	5.942	0
	Levodopa + Dopamine agonist	5.5351	0
	Dopamine agonist + Other medicine	3.8986	0
	Levodopa + Dopamine agonist + Other medicine	6.1054	0
${t}_{2}$	(Variables used in actions)		
	Levodopa	− 6.9801	0
	Dopamine agonist	− 5.0293	0
	Other medicine	− 0.825	0.094
	Levodopa + other medicine	− 6.9232	0
	Levodopa + Dopamine agonist	− 7.2501	0
	Dopamine agonist + other medicine	− 5.1182	0
	Levodopa + Dopamine agonist + other medicine	− 8.5083	0
	(Outcome) total UPDRS III scores		

We fitted prediction model as total UPDRS scores at ${t}_{2}$ (penalty) ~ variables at ${t}_{1}$ (used in disease state) + variables at ${t}_{2}$ (actions) using multivariate regression (i.e., generalized least square) and selected variables that are statistically significant on total UPDRS scores on the next timestep (${t}_{2}$). Note that the % change of total UPDRS scores are 100% $\times$ (Total UPDRS scores at ${t}_{1}$ − Total UPDRS scores at ${t}_{0}$)/Total UPDRS scores at ${t}_{0}$. AR = Akinetic-rigid, TD = tremor-dominant.

### Penalty

We used total UPDRS III scores at every visit as a penalty (or negative reward) of the medication management. We also added the number of medications taken to the penalty to avoid building drug tolerance by taking medicine not at critical timing. So the reward $r(s,a,s{^{\prime}})$ is the sum of (i) the expected total UPDRS III scores when choosing an action $a$ (i.e., the combination of drugs) at current disease state *s* and next disease state $s$’ and (ii) the number of medications in action $a$ multiplied by weighting constant $c$. We computed the expected reward from the patient’s trajectories.

As clinicians aim to decrease the penalty at the next visit and all subsequent visits, we introduce a weighting factor to balance the importance between current and future values. A discount factor $\gamma (0\le \gamma \le 1)$ determines how much weight (importance) is given to future penalties compared to the current instant penalty. For example, we can choose an initial amount, say $\gamma =3$ which means that we multiply ${0.3}^{1}$ to the penalty ${r}_{t+1}$ on next visit *t* + *1*, ${0.3}^{2}$ to ${r}_{t+2}$ on next visit *t* + *2*, and so on. Thus, the total future discounted penalty at visit $t$ is ${R}_{t}={\sum }_{{t}^{^{\prime}}=t+1}^{T}{\gamma }^{{t}^{^{\prime}}-t}{r}_{t{^{\prime}}}$, which $T$ is the final time-step at which the treatment ends.

### Transition probability

The actions (medication) change the states (disease states). So, we extracted transition probability from one state to another conditioned on an action using the patient’s trajectories. That is, the transition probability $T(s,a,s{^{\prime}})$ is the probability that, when a patient’s disease state is $s$ and the clinician decides to prescribe medication $a$ at current visit $t$, the patient’s disease state becomes $s{^{\prime}}$ at the next visit *t* + *1*. We computed the transition probability by counting each transition observed in the PPMI trajectory divided by the total number of transitions.

### Policy and value functions

RL involves estimating value function $v(s)$-a function that estimates how much total reward can be expected for an agent to be in a given state $s$*.* As the amount of total reward depends on the choice of actions, value functions are defined with respect to particular ways of acting, called policies^15^. A policy $\pi$ is a mapping from states to probabilities of selecting each possible action. For simplicity, the policy can be deterministic, *i.e.*, $a=\pi (s)$ that maps a state to action. The value ${v}_{\pi }(s)$ of a state $s$ under a policy $\pi$ is then expected the total amount of rewards when starting in $s$ and following $\pi$ thereafter*.* Similarly, we can define the value of taking action $a$ in a state $s$ under a policy $\pi$*,* denoted ${q}_{\pi }(s,a)$, where ${v}_{\pi }\left(s\right)={q}_{\pi }(s, \pi (s))$^15^.

### Estimation of AI’s and clinician’s policy

We derived a computationally optimal policy from the MDP and policy iteration, which we call ‘AI’s policy’${\pi }_{1}$^8^. We also evaluated the actual actions in the clinician’s practice using temporal difference learning, following the same evaluation framework of the previous study^8^.

A detailed formulation for policy iteration and temporal difference learning can be found in Supplement 1 and Supplement 2, respectively.

### Policy evaluation and comparison

Accurate evaluation of AI-driven policy is important when we want to know the value of the policy before it is deployed in real practice. We evaluated the value $V$ of the AI-driven policy ${\pi }_{1}$ using the actual trajectories of patients (generated by clinicians’ practice), which is so-called off-policy evaluation methods^16^. The off-policy evaluation aims to estimate the value of a new policy (target policy, e.g., AI policy) based on data collected by a different policy (behavior policy, e.g., clinicians’ practice). One of the most widely used off-policy evaluation approaches is importance sampling (IS), which corrects the mismatch between the distributions induced by the target policy and by the behavior policy^16,17^. Particularly, stepwise weighted importance sampling (step-WIS) is considered as the most practical point estimator in the importance of sampling techniques thanks to its low variance^18,19^. We also computed a confidence interval of policy values by bootstrapping trajectories^8^. Detailed formulation can be found in Supplement 3.

### Ensemble of policy

**Figure 2:**
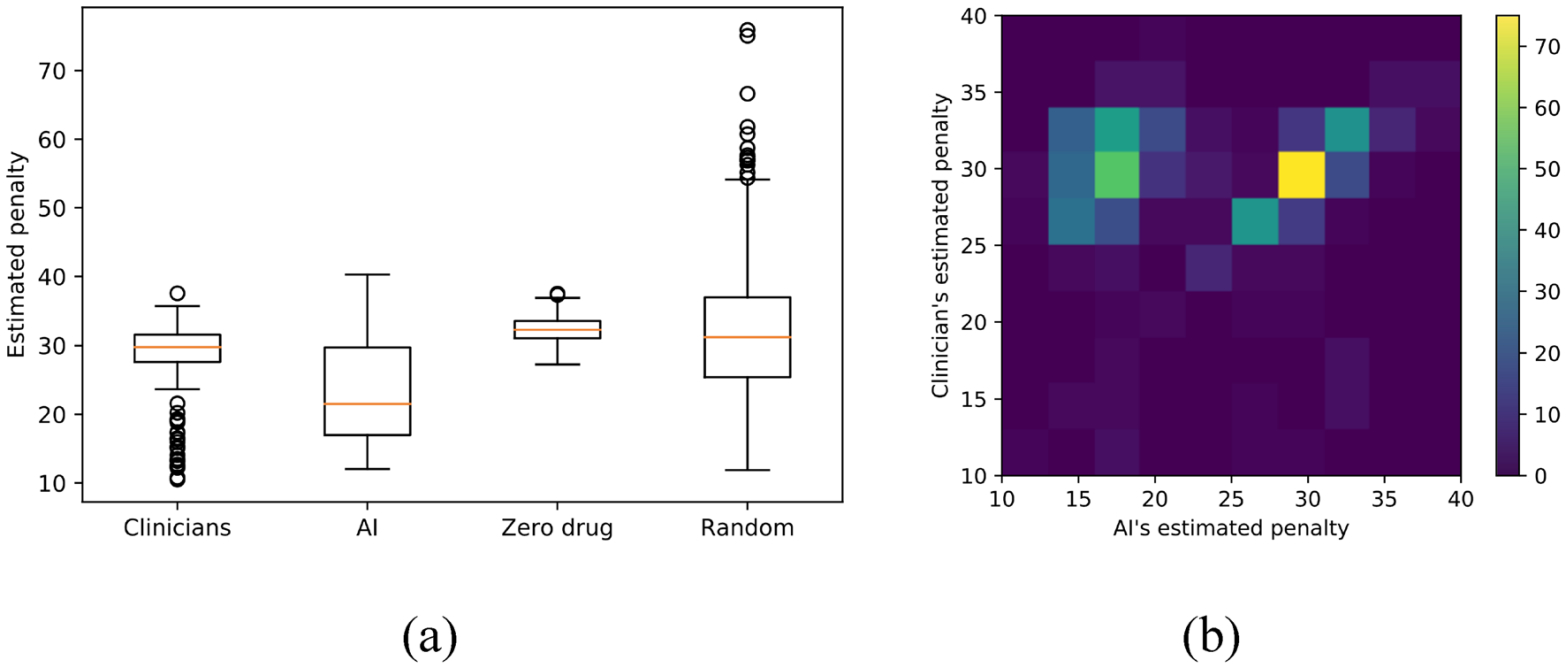
Comparison of estimated penalty distributions (i.e., the estimated cumulative sum of future total UPDRS III scores). The penalty scores of an individual patient is $V={\sum }_{t=1}^{T}{\gamma }^{t-1}{r}_{t}$ where ${r}_{t}$ is total UPDRS III scores at each visit with a discount factor $\gamma =0.3$. The estimate of $V$ was computed by importance sampling. We computed the penalty scores distribution across 500 independent bootstrapping with resampled training (80%) and test (20%) set. The final policy was chosen by the majority vote from 500 bootstraps. (**a**) Comparison of the penalty scores from four different strategies: clinicians AI’s policy, Zero drug (i.e., no drugs are given for all states), and random drug (i.e., any random drugs are given). Each box extends from the lower to upper quartile. A horizontal line in the box is a median. (**b**) Pairwise comparison of the penalty scores between AI and clinician.

We used an ensemble approach to derive a robust policy. We separate the patients into 80% training and 20% test set. After deriving both AI policy and clinician’s practice from training, we evaluated the estimated reward (or penalty) of the policy using the remaining 20% testing set as stepwise WIS. We randomly resampled the training and testing set 500 times and computed the distribution of estimated rewards (Fig. 2). The final optimal policy that maps a state to action was chosen by a majority vote from the 500 bootstraps.

## Results

We found that the RL approach automatically derives a medication regimen that is comparable to clinicians. We first confirmed that clinical assessment scores (for disease states) and medications (for actions) have enough statistical significance in predicting next UPDRS III scores (for the penalty) using a multivariate regression model (Table 2). We then derived 28 discrete disease states that regress to the next total UPDRS III scores using the statistically significant variables and decision tree regressor (Table 3). There were 21, 4, and 3 disease states for akinetic-rigid, tremor-dominant, and mixed type, respectively. The disease states mainly consisted of total UPDRS III scores, its change (between the previous and current values), and age, which clinicians can assess at every visit. The other variables (including cognition scores, Hoehn and Yahr disease stage) were statistically significant but less dominant in the decision tree regressor, thus did not use to define the disease states.

**Table 3:** Disease states and recommended actions in each state.

Disease state					Estimated Total UPDRS at ${t}_{2}$	Actions					
						clinicians			AI		
State	Subtype	Total UPDRS at ${t}_{1}$	% UPDRS change	Age		L	D	O	L	D	O
0	AR	( , 8]			10.2			×			×
1	AR	[9, 11]	[− 25.6, )		11.9		×				
2	AR	[12, 14]	[− 25.6, )		14.7		×	×	×	×	×
3	AR	[15,17]	[− 12.7, )		17.0		×			×	
4	AR	[9,14]	( , − 25.6]		17.1	×		×	×		×
5	AR	[18,19]	[− 12.7, )		19.3		×	×		×	×
6	AR	[20,24]	[16.2, )		20.0	×		×	×	×	
7	AR	[15,19]	( , − 12.7]		21.9		×	×	×	×	
8	AR	[20,24]	(− 14.6, 16.228]	[65, )	23.4	×	×	×	×	×	×
9	AR	[20,24]	(− 14.6, 16.228]	( , 64]	24.0	×	×			×	
10	AR	[25,33]	(39.6, )		25.1		×	×		×	×
11	AR	[20,24]	( , − 14.6]		26.6	×	×	×	×	×	×
12	AR	[25,28]	( − 7.28, 11.8]		27.5	×	×	×		×	
13	AR	[25,33]	( 11.8, 39.6]		27.7	×	×		×	×	
14	AR	[25,28]	( , − 7.28]		29.5	×	×	×	×		×
15	AR	[29,33]	(− 3.1, 11.8]		30.5	×	×	×	×	×	×
16	AR	[34,39]	(10.6, )		31.3	×	×	×	×	×	×
17	AR	[29,33]	( , − 3.1]		32.5	×	×	×	×	×	×
18	AR	[34,39]	( , 10.6]		36.8		×	×		×	×
19	AR	[40,45]			37.9	×	×	×		×	
20	AR	[46, )			46.5	×	×	×	×	×	×
21	Mixed	( ,16]			13.5		×	×	×		
22	Mixed	[17,24]			21.3	×	×	×	×		
23	Mixed	[25, )			32.1	×		×	×		×
24	TD	( , 14]			11.8		×			×	
25	TD	[15,19]			19.0	×		×	×	×	
26	TD	[20,28]			23.0	×		×	×		×
27	TD	[29, )			33.1	×	×	×	×	×	×

*L* Levodopa, *D* dopamine agonist, *O* other medicine, *AR* Akinetic-rigid, *TD* tremor-dominant.

Given the disease states, the reinforcement learning model derived optimized actions that can achieve the lowest median value of penalty (i.e., the cumulative sum of total UPDRS scores) (Fig. 2a). The median penalty value of clinician and AI were 29.8 and 21.5, respectively. Range and variance of the penalty values were [min = 10.5, max = 37.6, var. = 16.1] for clinicians and [min = 12.0, max = 40.3, var. = 47.4] for AI. These penalty distributions imply that AI’s medication policy achieves the lower penalty (i.e., fewer motor symptoms) in spite of the fact that the performance sometimes is not consistent, whereas clinicians achieve consistent but relatively high penalty (i.e., more motor symptoms). We confirmed the penalty values of AI are significantly lower than that of clinicians using a one-tail $t$-test (*t*-value = − 16.0, *p*-value = 1e−51). We also compared the penalty distribution of AI and clinicians as a one-to-one matching with the same training/test set and found that the penalty scores of AI are less or similar to that of clinicians in most cases (Fig. 2b). For comparison, we measured the penalty distribution of zero drugs (i.e., no drugs are given at all times) and random drugs (i.e., any random drugs are given at all times). We confirmed that medication policies of clinicians and AI achieve significantly lower penalty than the zero or random policy (*t*-value = − 16.7, *p*-value = 1e−55 for clinicians vs zero drugs; *t*-value = − 6.2, *p*-value = 1e−10 for clinicians vs random drugs).

**Figure 3:**
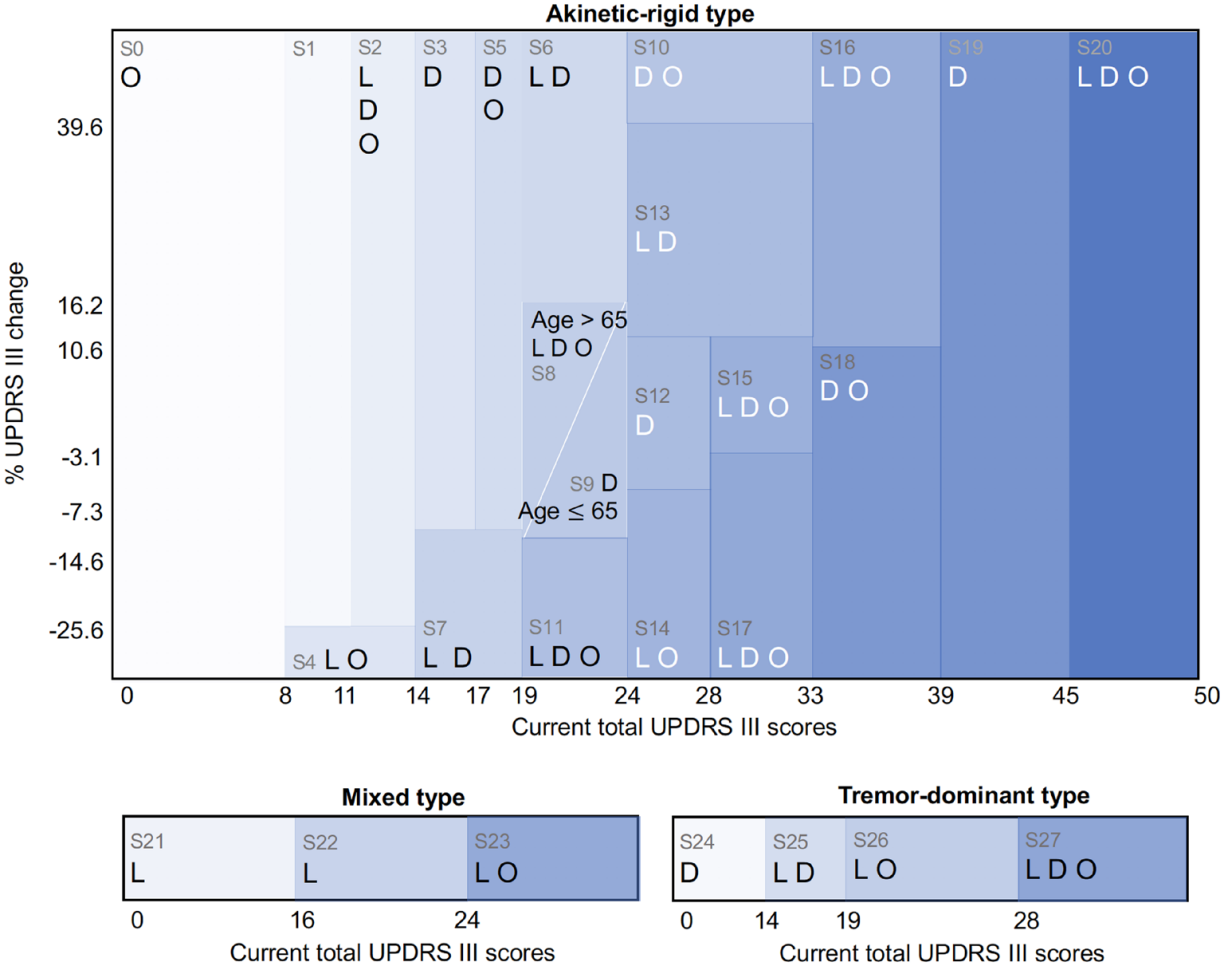
Disease states and suggested medications. Shade intensity is proportional to the estimated subsequent total UPDRS III scores. *L* levodopa, *D* dopamine agonist, *O* other medications.

We also compared the clinician’s suggestions and the recommended actions from AI (Table 3, Fig. 3). The AI model mostly followed clinicians but also suggested some changes in certain disease states. Among the 28 states, AI suggested the same actions with clinicians in 16 states and different actions in 12 states. For example, in the disease state 1, which is an early stage of akinetic-rigid PD with total UPDRS III scores between 9 and 11, clinicians usually prescribe dopamine agonists, whereas the AI model suggests holding off drugs. In disease state 9, which is a mild stage of akinetic-rigid PD with current total UPDRS III scores between 20 and 24 and age younger than 65, clinicians prescribe levodopa and dopamine agonists together, whereas the AI model suggests taking dopamine agonists alone.

## Discussion

The initial objective of this study was to show the effectiveness of RL to derive a robust and optimal combination of medication. Using an observational longitudinal cohort of PD patients, we derived clinically relevant disease states and an optimal combination of medication for each of them using policy iteration of the Markov decision process. As a result, RL derived a medication regimen that is comparable to a clinician’s decision. The AI model achieved a lower level of motor symptom severity scores than what clinicians did, whereas the clinicians’ medication rules were more consistent than the AI model. The AI model followed the clinician’s medication rules in most cases but also suggested some changes, which leads to the difference in lowering symptoms severity.

Our data-driven disease states were clinically relevant in terms of representing severity and accessible in an outpatient setting. The data-driven disease states were designed taking into consideration the most relevant patient-related factors for the clinical setting: (a) Age, which has important implications for tolerability of certain drugs; (b) Severity of symptoms, measured by the motor section of the UPDRS which also represents the level of disability, (c) Disease Stage, assessed by Hoehn and Yahr Scale, (d) Cognition, defined by the score on The Montreal Cognitive Assessment and finally, (e) PD motor subtype, which illustrates the rate of progression, in reference to the more rapid rate of deterioration exhibited by the akinetic-dominant subtype^20^. We incorporated clinician’s experiences into the definition of disease states by enforcing initial nodes to the PD motor subtype^2,3^, accordingly the disease states were highly interpretable and clinically relevant. The previous study on sepsis defines 750 disease states for an inpatient setting without prior knowledge of the disease^8^. This approach may not be directly applicable to the outpatient setting that does not have a vast amount of information flowing into the system, not to mention the inferior interpretability.

AI through RL provided a medication regimen comparable to clinicians, and although on average the outcome was better (lower UPDRS scores) there was variability in the estimated penalty compared to clinician’s penalty distribution (Fig. 2a), implying that the AI’s performance is not consistent enough across different trials. This result may be due to trial-and-error exploration in the action space (i.e., the combination of medications on each disease state). This inconsistent performance is also reported in the previous sepsis study^8^. To increase the robustness of AI’s decision, we aggregated the AI’s decisions from 500 bootstrapped trials into one regimen using a majority vote. This improved robustness is analogous to improved accuracy in ensemble strategies (e.g., random forest, gradient boosting). In addition, the accuracy of RL combined with the physician consistency clearly complement each other and potentially allow a better outcome for patients in terms of motor symptoms control.

Our study has several limitations. From a computational perspective, we encountered some negative values as the % UPDRS change which do not align with the normal progression of the disease. This might be due to inconsistent UPDRS III scores in the relatively small cohort size. Lack of external validation is another main limitation that reduces the generalizability of our results. From a clinical perspective, we failed to consider several important variables such as dosage and environmental factors affecting medication adherence.

## Conclusions

RL can enhance the pharmacological approach of PD patients by providing suggestions that efficiently and effectively address motor symptoms. This model will be extremely valuable for general neurologists and primary care physicians when they encounter complex cases in which there is reasonable doubt for the best combination of medication. This work is the beginning of the development of an interactive AI-physician ecosystem.

## Supplementary Information

Supplementary Information.

## Data Availability

Data used in the preparation of this article were obtained from the Parkinson’s Progression Markers Initiative (PPMI) database (www.ppmi-info.org/data). For up-to-date information on the study, visit www.ppmi-info.org.
