# Supplementary material for: Computational medication regimen for Parkinson’s disease using reinforcement learning
Source: Sci Rep. 2021 Apr 29;11:9313. doi: 10.1038/s41598-021-88619-4 (PMC8085228; doi:10.1038/s41598-021-88619-4)
Supplement: Supplementary file 1 — Supplementary Information. [file 41598_2021_88619_MOESM1_ESM.docx]

**Computational Medication Regimen**

**for Parkinson’s Disease using Reinforcement Learning**

**Authors:** Yejin Kim, Ph.D.^1^*, Jessika Suescun, M.D.^2^, Mya C. Schiess. M.D.^2^, Xiaoqian Jiang, Ph.D.^1^

*Supplement 1: Estimation of AI’s policy.*

We derived a computationally optimal policy from the MDP and policy iteration, which we call ‘AI’s policy’ $\pi_{1}$.[^1^](https://paperpile.com/c/Aec7ok/ShnB9) It consists of two steps: i) policy evaluation and ii) policy improvement. During the policy evaluation, $v(s)$is updated using dynamic programming:

$v_{\pi_{1}}(s)=\sum_{a} \pi_{1}(s,a)\sum_{s'} T(s,a,s')[r(s,a,s')+\gamma\cdot v_{\pi_{1}}(s')]$,

which starting from randomly assigned $\pi_{1}$. Then the policy can be improved by selecting greedily an action $a$ that give maximum value given state *s*:

$$\pi'_{1}(s)=argmax_{a}q_{\pi_{1}}(s,a)$$

In this way, we can derive optimal policy $\pi_{1}$that maps the best action given states as maximizing the total reward. Estimation of clinician’s actions can be found in Supplementary.

*Supplement 2: Evaluation of clinicians’ actions.*

We evaluated the actual actions in the clinician’s practice. We followed the same evaluation framework of the previous study.[^1^](https://paperpile.com/c/Aec7ok/ShnB9) We extracted policy $\pi_{0}$of the actual clinician’s practice from trajectories. This policy represents a clinician’s averaged medication action that we can observe from trajectories. Specifically, we used temporal difference learning (TD-learning), a model-free RL method [^2^](https://paperpile.com/c/Aec7ok/srMq) that directly learns policy from clinician’s practice without a predefined model of the medication dynamics. For all trajectories of actual clinician's practice, we iteratively updated the value function $q_{\pi}(s,a)$ on state $s$ and action $a$ as

$$q_{\pi_{0}}(s,a)\leftarrow q_{\pi_{0}}(s,a)+\alpha[r+\gamma\cdot q_{\pi_{0}}(s', a')-q_{\pi_{0}}(s,a)],$$

where $s'$ and $a'$is state and action of the next time step, respectively. $\alpha$ is updating constant and *r* is the reward given at current state and action. We repeated the updates with 500 random resampling of patient’s trajectories.

and derived optimal decision rules from policy iteration to minimize the cumulative sum of total UPDRS III scores with fewer medications. We separated the patient’s trajectory into an 80% training set and 20% testing set with 500 repeated random sampling. We used off-policy evaluation to estimate the value of the new policy we derived. We derived a robust policy using an ensemble of the 500 different policy. For hyper-parameters we set the weighting constant between total UPDRS III scores and the number of medications used in the penalty as 1, discount factor as 0.3 after extensive trials.

*Supplement 3: Policy evaluation and comparison.*

We define $\pi_{1}$as the target policy (to be evaluated) and $\pi_{0}$as behavior policy (by clinicians). The stepwise IS estimator provides an estimate of $\pi_{1}$’s value by multiplying the importance ratio in each time-step and averaging them:

$$V=\sum_{t=1}^{T} \gamma^{t-1}\rho_{1:t}r_{t}$$

where $\rho_{t}:=\pi_{1}(a_{t}|s_{t})/\pi_{0}(a_{t}|s_{t})$ is a per-step importance ratio, and $\rho_{1:t}:=\prod_{t'=1}^{t} \rho_{t'}$ is a cumulative importance ratio from the initial step to time-step $t$. Typically, this IS estimator suffers from high variance. The WIS is a variant of IS and relatively consistent. We define $w_{t}=\sum_{i=1}^{|D|} {\rho_{1:t}}^{(i)}/|D|$ as the average cumulative importance ratio at time-step $t$in a dataset $D$. The stepwise WIS is then

$V=\sum_{t=1}^{T} \gamma^{t-1}\frac{\rho_{1:t}}{w_{t}}r_{t}$,

and we averaged the estimate of the trajectory across all trajectories in $D$.

**References**

1. [Komorowski M, Celi LA, Badawi O, Gordon AC, Faisal AA. The Artificial Intelligence Clinician learns optimal treatment strategies for sepsis in intensive care. *Nat Med*. 2018;24(11):1716-1720.](http://paperpile.com/b/Aec7ok/ShnB9)

2. [Sutton RS. Introduction: The Challenge of Reinforcement Learning. In: *Reinforcement Learning*. ; 1992:1-3.](http://paperpile.com/b/Aec7ok/srMq)
